# High resolution melting curve analysis enables rapid and reliable detection of G6PD variants in heterozygous females

**DOI:** 10.1186/s12863-018-0664-1

**Published:** 2018-08-10

**Authors:** Md Tarikul Islam, Suprovath Kumar Sarker, Shezote Talukder, Golam Sarower Bhuyan, Asifuzzaman Rahat, Nafisa Nawal Islam, Hasan Mahmud, Mohammad Amir Hossain, A. K. M. Muraduzzaman, Jakia Rahman, Syeda Kashfi Qadri, Mohammod Shahidullah, Mohammad Abdul Mannan, Sarabon Tahura, Manzoor Hussain, Narayan Saha, Shahida Akhter, Nazmun Nahar, Firoza Begum, Tahmina Shirin, Sharif Akhteruzzaman, Syed Saleheen Qadri, Firdausi Qadri, Kaiissar Mannoor

**Affiliations:** 1 Laboratory of Genetics and Genomics, Institute for Developing Science and Health Initiatives, Mohakhali, Dhaka, Bangladesh; 2 Infectious Diseases Laboratory, Institute for Developing Science and Health Initiatives, Mohakhali, Dhaka, Bangladesh; 3 Department of Virology, Institute of Epidemiology, Disease Control and Research, Mohakhali, Dhaka, Bangladesh; 4 Department of Paediatric Medicine, KK Women’s and Children’s Hospital, Singapore, Singapore; 5 Department of Neonatology, Bangabandhu Sheikh Mujib Medical University, Shahbag, Dhaka, Bangladesh; 6 Department of Paediatric hematology and oncology, Dhaka Shishu Hospital, Dhaka, Bangladesh; 7 Department of Paediatric Medicine and Cardiology, Dhaka Shishu Hospital, Dhaka, Bangladesh; 8 Department of Paediatric Neurology, National Institute of Neurosciences & Hospital, Dhaka, Bangladesh; 9 Department of Paediatrics, Bangladesh Institute of Research & Rehabilitation in Diabetes, Endocrine and Metabolic Disorders, Shahbag, Dhaka, Bangladesh; 10 Department of Obstetrics and Gynecology, Bangabandhu Sheikh Mujib Medical University, Dhaka, Bangladesh; 11 Department of Genetic Engineering & Biotechnology, University of Dhaka, Dhaka, Bangladesh; 12 Department of Enteric and Respiratory Infectious Diseases, Infectious Diseases Division, International Centre for Diarrhoeal Disease Research, Bangladesh, Dhaka, Bangladesh

**Keywords:** Glucose-6-phosphate dehydrogenase deficiency, Heterozygous G6PD variants, G6PD heterozygosity, High resolution melting curve analysis

## Abstract

**Background:**

Like glucose-6-phosphate dehydrogenase (G6PD) deficient hemizygous males and homozygous females, heterozygous females could also manifest hemolytic crisis, neonatal hyperbilirubinemia or kernicterus upon exposure to oxidative stress induced by certain foods such as fava beans, drugs or infections. Although hemizygous males and homozygous females are easily detected by conventional G6PD enzyme assay method, the heterozygous state could be missed by the conventional methods as the mosaic population of both normal and deficient RBCs circulates in the blood. Thus the present study aimed to apply high resolution melting (HRM) curve analysis approach to see whether HRM could be used as a supplemental approach to increase the chance of detection of G6PD heterozygosity.

**Results:**

Sixty-three clinically suspected females were evaluated for G6PD status using both enzyme assay and HRM analysis. Four out of sixty-three participants came out as G6PD deficient by the enzyme assay method, whereas HRM approach could identify nine participants with G6PD variants, one homozygous and eight heterozygous. Although only three out of eight heterozygous samples had G6PD enzyme deficiency, the HRM-based heterozygous G6PD variants detection for the rest of the samples with normal G6PD enzyme activities could have significance because their newborns might fall victim to serious consequences under certain oxidative stress.

**Conclusions:**

In addition to the G6PD enzyme assay, HRM curve analysis could be useful as a supplemental approach for detection of G6PD heterozygosity.

## Background

Glucose-6-phosphate dehydrogenase (G6PD) deficiency is an X-linked inherited disorder with a worldwide prevalence of 4.9% and the disorder affects 400 million people globally [1]. The G6PD gene-encoded enzyme is involved in the production of NADPH, which maintains RBCs’ reduced glutathione levels and consequently plays a role in keeping cellular proteins and lipids in the reduced state when erythrocytes are subjected to an oxidative stress [2]. Most individuals with G6PD deficiency remain clinically asymptomatic. However, a reduction in G6PD enzyme activity makes RBCs susceptible to hemolysis under conditions of oxidant drug administration, ingestion of foods which induce oxidative stress, or infections [3, 4].

As an X-linked genetic disorder, G6PD deficiency is more likely to affect males than females. The major clinical manifestations are generally noticed in hemizygous males and homozygous females [5–8]. However, studies have shown that random X-chromosome inactivation can result in mosaic populations of normal and deficient erythrocytes [9] and heterozygous females can also be affected in some circumstances and especially such a situation can occur when the population of deficient RBCs develops hemolysis under conditions of oxidative stress [10–12]. Thus, G6PD heterozygous females are also susceptible to oxidative stress-induced hemolysis, even though the severity is variable. The most serious outcome might be seen in the heterozygous newborns who could suffer from an acute hemolytic crisis resulting in an acute bilirubin-induced encephalopathy, kernicterus and even death [13–15]. Moreover, it has been illustrated that the proportion of defective to normal RBC population might be subjected to change over time, e.g., the age-related bias of X-chromosome inactivation leading to G6PD deficiency in octogenarian, nanogenarian, and centenarian females in a population with prevalent G6PD variants have been reported [16]. In some cases, the numbers of defective RBCs are much higher and total G6PD enzyme activity might be comparable to those in G6PD deficient hemizygous males. In a situation like this, hemolysis is inevitable upon exposure to oxidant drugs, fava beans or infectious agents. Under the circumstances, knowledge of heterozygous polymorphic status in the G6PD gene of females would help the concerned persons to avoid certain foods and drugs that may make heterozygous females victims to oxidative stress. Additionally, heterozygous females could give birth to hemizygous male newborns who could suffer from hemolytic crisis upon accidental exposure to oxidative stress which could lead to hyperbilirubinemia, kernicterus and even death [17, 18].

Although typical screening tests detect hemizygous G6PD deficient males and homozygous G6PD deficient females with ease, they do not detect heterozygous females with high efficiency. The fluorescent spot test and Quantitative G6PD enzyme assay methods are based on the principle of measurement of NADPH produced from NADP^+^ by G6PD enzyme. So, these conventional methods are likely to misdiagnose G6PD heterozygous females with a higher proportion of normal to deficient RBCs population in the circulation [19, 20]. On the other hand, although the cytochemical assay and MRT-based cytofluorometric method could differentiate among G6PD normal, G6PD hemizygous, G6PD homozygous and G6PD heterozygous samples, these methods are cumbersome, requiring several steps, prone to error, and it is difficult to process and screen a large number of samples using these approaches [19, 21]. The DNA-based tests are also reliable and can be used for diagnosis of patients with homozygous, hemizygous, and heterozygous G6PD deficiency. But most of the DNA-based methods, such as sequencing, denaturing high-performance liquid chromatography (DHPLC), amplification refractory mutation system polymerase chain reaction (ARMS-PCR), single strand conformational polymorphism (SSCP) etc. are either costly or technically cumbersome and requires the use of hazardous chemical compounds. Thus, in addition to the quantitative G6PD enzyme assay, a supplemental DNA-based rapid and reliable approach such as high-resolution melting (HRM) curve analysis for screening of G6PD variants could be really useful to increase the chance of detection of heterozygous status.

High-resolution melting (HRM) curve analysis is a DNA-based high throughput, rapid and reliable mutation screening approach that can effectively separate wild-type status from hemizygous, homozygous and heterozygous statuses [22]. The approach has successfully been applied for screening of genetic variants involved in various genetic diseases including autosomal recessive, autosomal dominant and X-linked recessive disorders [23–25]. This present study aimed to apply HRM curve analysis approach to see whether HRM could be used as a supplemental approach to increase the rate of detection of G6PD heterozygosity.

## Results

### Demographic information and G6PD enzyme status of the study participants

Depending on the history of the previous hemolytic crisis, all sixty-three Bangladeshi Bengali female participants who were in the age range of 0–15 years were suspected of having G6PD deficiency. The mean age of the study participants were 8.47 ± 3.24 years. To diagnose how many of the suspected participants had an actual G6PD deficiency, quantitative enzyme assay was performed using RBC specimens of each participant. Among 63 participants, only 4 (6.34%) came out as G6PD enzyme deficient, provided that the cut-off for G6PD enzyme deficiency was set to 7.37 U/g Hb, as demonstrated in our previous study [26].

### HRM-based assessment of samples targeting the common G6PD variants in Bangladesh

**Fig. 1:**
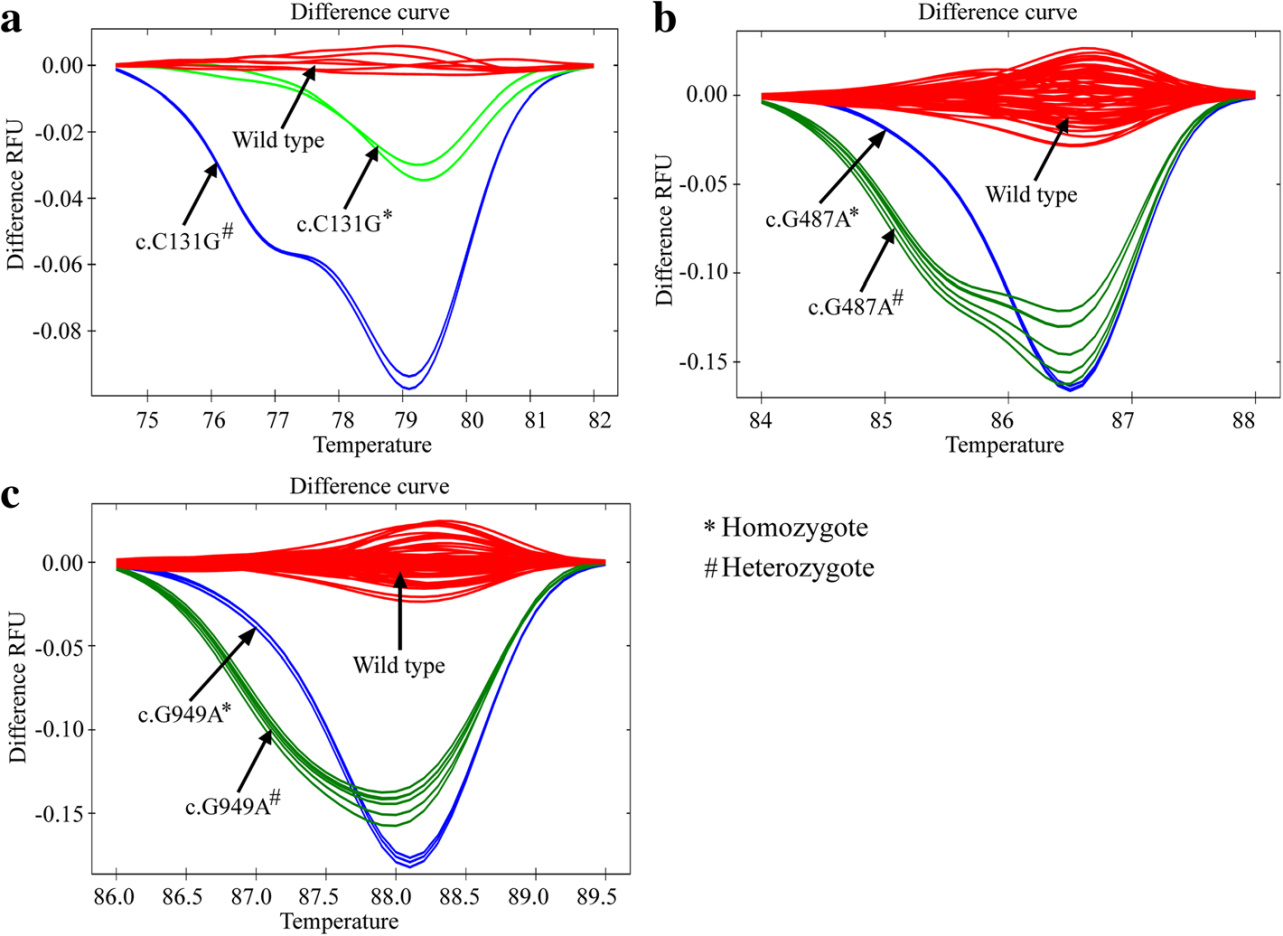
HRM curves patterns for the indicated common mutations in the glucose-6-phosphate dehydrogenase gene for the study participants to differentiate homozygous and heterozygous states from each other and from the wild-type alleles. (**a**) G6PD Orissa mutation, (**b**) G6PD Mahidol mutation and (**c**) G6PD Kalyan-Kerala mutation in homozygous or heterozygous states which could be unambiguously distinguished from the wild-type alleles

All 63 specimens were subjected to HRM analysis targeting three previously reported G6PD common variants in Bangladesh [26]. HRM curve analysis could identify 7 out of 63 samples that were found to vary from the wild-type alleles (Fig. 1a, b, and c). The HRM curve results were confirmed by Sanger sequencing of all samples. None of the samples had SNPs or G6PD variants that clustered with the wild type reference samples. Three out of seven samples that had deviated from wild type allele were found to have the enzyme activities lower than the cut-off (3.1 U/g Hb, 4.89 U/g Hb, and 5.45 U/g Hb), whereas the enzyme activities of the remaining four samples were greater than the cut off (7.67 U/g Hb, 9.38 U/g Hb, 10.84 U/g Hb, and 11.34 U/g Hb) (Table 1).

**Table 1:** G6PD enzyme activity in the samples with G6PD Orissa, G6PD Mahidol, and G6PD Kalyan-Kerala variants

Mutations	Exons	Amino acid substitution	Enzyme activity U/g Hb	Enzyme activity compared to the cut-off (%)
c.C131G^*^	Exon 3	p. Ala44Gly	3.10	57.93% ↓
c.C131G^#^	Exon 3	p. Ala44Gly	4.89	33.60% ↓
c.G487A^#^	Exon 6	p. Gly163Ser	7.67	03.91% ↑
c.G487A^#^	Exon 6	p. Gly163Ser	11.34	35.04% ↑
c.G949A^#^	Exon 9	p. Glu317Lys	5.45	26.03% ↓
c.G949A^#^	Exon 9	p. Glu317Lys	9.38	21.45% ↑
c.G949A^#^	Exon 9	p. Glu317Lys	10.84	33.99% ↑

# indicates heterozygous variants; *indicates homozygous variants; ↓ indicates lower; ↑ indicates higher

The sequencing data showed that the Orissa variant was present in a homozygous state in one sample with an enzyme activity of 3.1 U/g Hb, whereas the same variant was found in one of the seven samples in a heterozygous state with an enzyme activity of 4.89 U/g Hb (Table 1). The sample with an enzyme activity of 5.45 U/g Hb was found to have G6PD Kalyan-Kerala (c.G949A) variant in a heterozygous state (Table 1).

Sequencing results also confirmed that four samples with enzyme activities greater than the cut-off but varied from the wild-type samples had genetic polymorphisms. All four of them were found to have heterozygous G6PD variants. Two of the samples had G6PD Mahidol (c.G487A) variant in a heterozygous state and their enzyme activities were 7.67 U/g Hb and 11.34 U/g Hb, respectively, whereas the other two samples with G6PD Kalyan-Kerala heterozygous variant had enzyme activities of 9.38 U/g Hb and 10.84 U/g Hb, respectively. Overall, the findings suggest that screening of G6PD variants by HRM curve analysis targeting the commonly occurring variants in Bangladesh can be performed with 100% sensitivity and 100% specificity.

### HRM-based assessment of samples targeting the G6PD variants other than common mutations

**Fig. 2:**
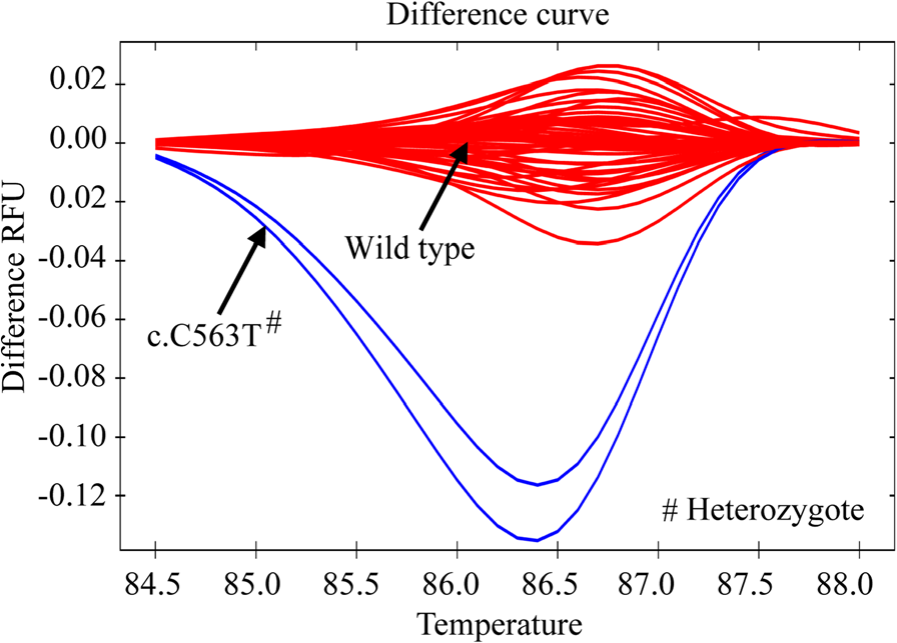
Identification of G6PD Mediterranean (c.C563T) mutations in a heterozygous state by using primers targeting exon-6. The HRM curve pattern for c.C563T heterozygous mutation could be distinguished from wild-type allele

When all 63 samples were subjected to HRM analysis to screen other unreported variants, only two samples were found to vary from the wild-type alleles (Fig. 2). Upon Sanger sequencing, both of these two samples were found to contain G6PD Mediterranean variant (c.C563T) in heterozygous states with enzyme activities of 3.52 U/g Hb and 11.89 U/g Hb (Table 2), respectively. The samples that made melt curve clusters with the wild type reference samples had no other G6PD variants, implying 100% sensitivity and specificity of the method. Thus, like the G6PD common variants, G6PD Mediterranean variants could also be targeted by HRM to detect G6PD heterozygote states in Bangladesh.

**Table 2:** G6PD enzyme activities in samples with the heterozygous G6PD Mediterranean variants

Mutations	Exons	Amino acid substitution	Enzyme activity U/g Hb	Enzyme activity compared to cut off (%)
c.C563T^#^	Exon 6	p. Ser188Phe	3.52	52.19% ↓
c.C563T^#^	Exon 6	p. Ser188Phe	11.89	38.02% ↑

^#^indicates heterozygous variants; ↓ indicates lower; ↑ indicates higher

### Skewing of G6PD enzyme activities in heterozygous female participants

The results described so far indicated that 8 out of 63 female specimens had heterozygous variants. Next, we wanted to investigate how the G6PD enzyme activities did vary among samples with different heterozygous states and within different samples of the same heterozygous states. Tables 1 and 2 show that samples with heterozygous variants had different levels of G6PD enzyme activities. Even samples with the same heterozygous variants differed greatly among one another in levels of G6PD enzyme activities. One of the eight samples with the heterozygous state had c.C131G variant and its enzyme activity was 4.89 U/g Hb, which was 33.60% lower than the cut-off 7.37 U/g Hb (Table 1). However, the enzyme activities of two of the samples with c.G487A heterozygous variant differed greatly, such as 7.67 U/g Hb and 11.34 U/g Hb, which were 03.91% and 35.04% higher than the cut-off (Table 1), respectively. Similar to the c.G487A heterozygous variant, two heterozygous c.C563T samples showed remarkable differences in their G6PD enzyme activities. One of these two samples had an enzyme activity of 3.52 U/g Hb, which was 52.19% lower than the cut-off, whereas the other one had 38.02% higher enzyme activity than the cut-off (Table 2). Finally, the study identified 3 samples with c.G949A heterozygous variant. One of these three samples had an enzyme activity of 5.45 U/g Hb, which was 26.03% lower than the cut-off. However, the enzyme activities of the rest two samples with c.G949A heterozygous variant were 9.38 U/g Hb and 10.83 U/g Hb, which were 21.45% and 33.99% higher than the cut-off (Table 1), respectively.

## Discussion

This present study demonstrates the use of high resolution melting (HRM) curve analysis as a supplemental approach in addition to G6PD enzyme assay to increase the rate of chance of detection of G6PD heterozygote females targeting the previously reported and unreported G6PD genetic variants in Bangladeshi population. Extensive genetic researches have been revealing huge useful information about genetic abnormalities including chromosomal aberration, overexpression of genes, or mutations of bases. Sensitive, accurate and timely identification of some SNPs or genetic variants can influence clinical decision making, such as in prescribing personalized medicine that may potentially lead to better prognosis. In some cases, SNPs or genetic variants detection may help to avoid disease-specific conditions, thereby offering an appropriate patients’ management strategy. However, it is really important to find a genetic variants detection method that is reliable, time-saving, and cost-effective and uses a high throughput operational platform to supplement the conventional methods which may be good for diagnosis but they have limitations with higher failure rates in specific conditions. In most cases, it is usual to target the gene-encoded products for the diagnosis of a genetic disease, such as fluorescent spot test or enzyme activity measurement for G6PD deficiency detection, as it is less cumbersome, cost-effective and quick. Unfortunately, targeting the gene-encoded products sometimes do not offer the real state of the disease and such an approach can lead to an unexpected clinical outcome. Such a situation could occur in females with heterozygous G6PD variants as skewed X-chromosome inactivation can result in a mosaic population of normal and deficient RBCs, where the levels of G6PD enzyme activity in heterozygote females depend on the ratio of G6PD normal to G6PD deficient erythrocytes [21, 27]. Thus, the present study aimed to illustrate the importance of a supplemental approach, known as high-resolution melting (HRM) curve analysis, in addition to G6PD enzyme assay method to accurately detect heterozygous females targeting the previously reported and unreported G6PD variants in Bangladesh.

In the present study, quantitative spectrophotometric G6PD enzyme assay method detected only 4 out of 63 samples with enzyme activities lower than the cut-off. On the other hand, HRM-based genetic testing that conformed to the Sanger sequencing data revealed that 9 of the 63 participants were carrying G6PD gene variants that cause G6PD deficiency, one in homozygous state and eight in heterozygous states. Five of the heterozygote samples had enzyme activities that were higher than the cut-off and such a finding could be attributed to the skewed X-chromosome inactivation which could result in a higher number of G6PD normal RBCs and a fewer G6PD deficient RBCs [21]. Even though these heterozygote females were normal in terms of G6PD enzyme activities, their enzyme activity status might alter due to a change in the ratio of the mosaic population of normal to deficient RBCs in the later stages of life [16]. Thus, these G6PD heterozygous females with normal enzyme activities might be vulnerable to suffer from oxidative stress upon exposure to oxidizing drugs and intake of certain food that induce oxidative stress. Prescription of oxidizing drugs to patients is generally done after a biochemical assay for G6PD in a population with a high frequency of G6PD deficiency. Once the test is done for G6PD deficiency and status of the patient is known, further checking of G6PD enzyme status might not be necessary during future treatment for a parasitic infection, such as for malaria infection. As the G6PD enzyme activity might be subjected to change to a low level due to alteration in a ratio of normal to deficient RBCs population, these heterozygous females are at risk of accidental G6PD deficiency-related complications [16]. Moreover, recent studies have demonstrated that antimalarial drugs can cause hemolysis of defective RBC population in heterozygous females [28]. Thus, it is important to know the G6PD heterozygous status and once it is known, avoidance of accidental hemolytic crisis in heterozygote females is possible.

Detection of G6PD heterozygosity is important not only for a deficient female but also for her newborn babies. It is important to take into consideration of the point that 400 million people are affected globally with G6PD deficiency [1]. Even though most of those affected people are males, but the important point to note is that those affected males inherit G6PD deficiency from their heterozygous or homozygous G6PD deficient mother. Thus, if the status of a mother is known it would be helpful for her to avoid accidental complications as well as to avoid neonatal problems that are more severe and most damaging. A heterozygous G6PD deficient mother who is never symptomatic of her deficiency may deliver a baby boy with the deficient allele. In such a situation, the baby boy with G6PD mutant allele may remain asymptomatic for the first few days of life and then may start showing symptoms of jaundice which may gradually worsen to kernicterus or bilirubin encephalopathy or even death [17, 18]. A female newborn with heterozygous G6PD deficiency has also been reported with similar features. This female newborn had a normal level of bilirubin for the first two days after birth and then routine screening revealed hyperbilirubinemia even though the G6PD enzyme activity was in the normal range [14]. Such a phenomenon was the result of the destruction of G6PD deficient RBCs, although the normal RBC population survived the oxidative stress. The potential damage due to G6PD heterozygous mutation does not end here. Extreme hyperbilirubinemia and death were accounted for a G6PD heterozygous female neonate who was also a heterozygote due to (TA)6/(TA)7 promoter polymorphism for uridine diphosphate glucuronosyltransferase 1A1*28 (UGT1A1*28) [13], which is generally not a risk factor for neonatal jaundice or kernicterus [29–31]. But UGT1A1*28 promoter polymorphism along with G6PD deficiency can lead to extreme neonatal hyperbilirubinemia [32].

Till date, at least 160 G6PD gene variants are known and these genetic variants are dispersed throughout the G6PD gene [33]. So, HRM curve analysis targeting the full-length G6PD gene is not feasible for large-scale screening. Fortunately, all of these G6PD variants are not present in all geographical locations of the world and only a small fraction of G6PD variants could be found for a specific geographical location and ethnic population. For example, only 9 variants, namely c.A95G, c.G392 T, c.G487A, c.A493G, c.C592T, c.C1024T, c.C1360T, c.G1376 T, and c.G1388A account for approximately 90% of G6PD deficiency in China [22, 34, 35]. Similarly, in Thai-Myanmar border area, only G6PD Mahidol (G487A) variant accounts for 88–96% of G6PD-deficient subjects [36, 37]. Three G6PD variants, namely G6PD Orissa, G6PD Mediterranean, and G6PD Kalyan-Kerala are commonly found in Indian population [38, 39]. G6PD Mediterranean (c.C563T) is also common in Pakistan and Afghanistan and accounts for approximately 80% of G6PD deficient cases in Pakistan [40, 41]. Thus, targeting a set of G6PD variants specific to the population of certain geographical location and ethnic background, HRM melting curve approach could be implemented for the screening of heterozygous G6PD variants. Hence, we planned to use HRM approach for screening of G6PD heterozygous females in Bangladesh because only three G6PD variants in the G6PD gene had been reported as the most common in the country [26]. In addition to three commonly occurring mutations, we had identified another G6PD variant, namely G6PD Mediterranean (c.C563T). Thus, only four sets of G6PD gene-specific HRM PCR primers could help avoiding misdiagnosis of G6PD heterozygous females.

In this study, we designed one set of primers for detection of G6PD Orissa (c.C131G) mutation and used a new combination of primers from previously published study for detection of G6PD Kalyan-Kerala variant (c.G949A) [22]. Additionally, for detection of mutations including c.A95G, c.C274T, c.G392 T, C406T, c.G487A, c.A493G, c.T517C, c. C519G, c.C592T, c.A835G, c.G871A, c.C1024T, c.C1004T, c.G1340 T, c.C1360T, c.G1376 T, c.G1381A and c.G1388A, published primers were used [22]. However, in the aforementioned study, the authors analyzed specimens of already known G6PD deficient persons and their parents. In this study, we used specimens of unknown G6PD enzyme statuses and identified some heterozygous samples that had normal G6PD enzyme activities. Moreover, we had illustrated the use of primers from the above-mentioned study to detect mutations like G6PD Kalyan-Kerala (c.G949A) and G6PD Mediterranean (c.C563T), which had not been reported by the authors of the cited article. Thus our HRM approach could screen G6PD variants in heterozygous females with 100% sensitivity and 100% specificity in Bangladesh. Most importantly, we had illustrated the importance of supplemental HRM approach in addition to quantitative enzyme assay method to maximize the chance of detection of G6PD heterozygous females.

## Conclusions

In summary, using conventional enzyme assay method a significant number of heterozygote females could be missed. G6PD enzyme activities vary greatly not only among the samples with different heterozygous genetic variants but also among the samples with same heterozygous G6PD genetic variants. A supplemental HRM approach targeting regional common G6PD variants could increase the chance of G6PD heterozygous females’ detection. Since G6PD variants repertoires differ among different geographical locations and ethnic populations, primer designing should be done accordingly.

## Methods

### Study participants

Based on their family history or past clinical hemolytic complications, sixty-three female participants of Bangladeshi Bengali ethnic origin in the age range of 0–15 years were enrolled at the clinical settings of Bangabandhu Sheikh Mujib Medical University; Bangladesh Institute of Research & Rehabilitation in Diabetes, Endocrine and Metabolic Disorders; and Dhaka Shishu Hospital. The participants either belonged to a family with one or more G6PD deficient case(s) or had a history of recovery from an episode of neonatal hyperbilirubinemia or jaundice or hemoglobinuria accompanied by pallor or an accidental hemolytic crisis. It was made sure that the participants who had a history of the previous hemolytic crisis were in remission for a sufficient period of time so that the factors that could affect G6PD enzyme assay could be avoided, such as higher G6PD enzyme activity after recovery from a hemolytic crisis. The Ethical approval for this study was taken from Bangladesh Medical Research Council (BMRC) of National Ethics Review Committee (NERC), Dhaka, Bangladesh. Prior to enrollment of the study participants, written informed consents were obtained from the parents or guardians of the study participants.

### Sample collection

One mL blood was collected by venipuncture from each of the participants in an ethylenediaminetetraacetate (EDTA)-coated vacutainer. A fraction of the collected blood was used for G6PD enzyme assay, whereas another fraction was used for genomic DNA extraction.

### Quantitative G6PD activity assay

Quantitative G6PD enzyme activity measurement was performed using Randox G6PD assay kit (Randox Laboratories Ltd., Crumlin, UK) and manufacturer’s instruction was followed. The enzyme activity assay was performed by measuring an increase in absorbance at 340 nm which is associated with an increase in NADPH concentration produced in the reaction catalyzed by G6PD enzyme. G6PD enzyme activity at ambient temperature was calculated at ~ 25 °C following the equation provided with the kit, (33,650 x ΔA 340 nm/min × 100) / Hb (g/dL), where ΔA means a change in absorbance per minute. The calculated value at 25 °C was multiplied by temperature correction factor 2.076 to get the G6PD enzyme activity at 37 °C.

### Extraction of genomic DNA from whole blood

Genomic DNA isolation from whole blood was performed using QIAGEN flexigene® DNA kit (Qiagen, Hilden, Germany) following manufacturer’s instructions.

### Real-time PCR-high resolution melting curve analysis

The real-time PCR was followed by HRM analysis and the procedures were performed in a CFX96 Touch™ Real-Time PCR machine (BioRad). The screening was performed targeting three reported G6PD variants in Bangladesh, namely G6PD Orissa (c.C131G), G6PD Mahidol (c.G487A) and G6PD Kalyan-Kerala (c.G949A) [26] and other unreported G6PD variants including c.A95G, c.C274T, c.G392 T, C406T, c.A493G, c.T517C, c. C519G, c.C563T, c.C592T, c.A835G, c.G871A, c.C1024T, c.C1004T, c.G1340 T, c.C1360T, c.G1376 T, c.G1381A and c.G1388A. For screening of G6PD Orissa (c.C131G) variant, PCR amplification was done using forward primer 5ˊ-CACCTGTTCCCTCTGCCAC-3ˊand reverse primer 5ˊ-TACCAGATGGTGGGGTAGATC-3ˊ which spans a 62 bp amplicon of G6PD gene. On the other hand, G6PD Kalyan-Kerala variant was screened by targeting a 226 bp sequence of G6PD gene using the forward primer 5ˊ-CCCAACTCAACACCCAAGGA-3ˊ and the reverse primer 5ˊ-CTCATTCTCCACATAGAGGACGAC-3ˊ [22]. For screening of other G6PD variants including c.A95G, c.C274T, c.G392 T, C406T, c.G487A, c.A493G, c.T517C, c. C519G, c.C563T, c.C592T, c.A835G, c.G871A, c.C1024T, c.C1004T, c.G1340 T, c.C1360T, c.G1376 T, c.G1381A and c.G1388A, PCR amplification was performed using previously published primers [22]. The lengths of the amplified product ranged from 62 bp to 226 bp, which were within the limit of standard amplicon size for HRM analysis [42, 43].

A reaction volume of 10 μL was used for real time-polymerase chain reaction (RT-PCR). The composition of PCR mixture was as follows: 5 μL of 2X precision melt supermix (BioRad), 0.2 μL of forward primers (10 μM), 0.2 μL of reverse primers (10 μM), 50 ng of genomic DNA in a total of 10 μL reaction volume. The thermal cycling profile for the real-time PCR was as follows: initial denaturation at 95 °C for 3 min; 40 cycles of denaturation at 94 °C for 10 s, annealing at 58 °C for 30 s and extension at 72 °C for 30 s. After completion of real-time PCR, the subsequent melt curve program had the following steps: denaturation at 95 °C for 30 s, renaturation at 60 °C for 30 s, and then melting at 60 °C to 95 °C with an increment of 0.1 °C per 5 s. Real-time HRM analysis was performed by parallel testing of (a) specimens of the present study, (b) sequenced wild type reference samples, and (c) available homozygous/hemizygous samples for G6PD Orissa (c.C131G), G6PD Mahidol (c.G487A) and G6PD Kalyan-Kerala (c.G949A) from our previous study [26].

### Polymerase chain reactions, PCR product purification, and sequencing

For confirmation of the HRM melt curve results, Sanger sequencing was performed for all samples as it has been reported that some G6PD variants might generate melting patterns that are similar to those generated by the wild type allele [44, 45]. Sequencing of G6PD gene was done using our previously published primers [26]. For sequencing, the extracted DNA samples were subjected to polymerase chain reaction (PCR) following aforementioned study protocol. Following completion of reaction cycles, PCR product purification was performed using the MinElute® PCR purification kit (Qiagen) according to manufacturer’s instruction.

The purified PCR product was subjected to chain termination reaction for Sanger sequencing using the Big Dye Version 3.1 Cycle Sequencing Kit (Applied Biosystems, Warrington, UK) following manufacturer’s instruction. Thereafter, the chain termination product was purified using the BigDye® XTerminator™ purification kit (Applied Biosystems). Finally, the purified chain termination PCR product was subjected to capillary sequencing in an ABI PRISM 310 Automated Sequencer (Applied Biosystems).

### Sequencing data collection and mutation identification

Sequencing data were collected by ABI PRISM 310 data collection software version 3.1.0 (Applied Biosystems). The collected data were exported in FASTA format and thereafter the obtained data were analyzed to identify G6PD variants by using Basic Local Alignment Search Tool (BLAST), which compared the query sequence with the reference (wild-type) sequence (NM_001042351.2) retrieved from NCBI database.

## Abbreviations

ARMS-PCR: Amplification refractory mutation system polymerase chain reaction
DHPLC: Denaturing high performance liquid chromatography
EDTA: Ethylenediaminetetraacetate
G6PD: glucose-6-phosphate dehydrogenase
HRM: High resolution melting
PCR: Polymerase chain reaction
RBCs: Red blood cells
SSCP: Single strand conformational polymorphism
UGT1A1: Uridine diphosphate glucuronosyltransferase 1A1
